# Sub-national disparities in accessing anti-malarial drug treatment in eastern Indonesia

**DOI:** 10.1186/s12889-021-11602-1

**Published:** 2021-08-13

**Authors:** Mara Ipa, Agung Dwi Laksono, Endang Puji Astuti, Heni Prasetyowati, Firda Yanuar Pradani, Joni Hendri, Andri Ruliansyah, Henry Surendra, Iqbal R. F. Elyazar

**Affiliations:** 1 Pangandaran Unit for Health Research and Development, National Institute of Health Research and Development, Ministry of Health of Indonesia, Pangandaran, West Java Indonesia; 2 National, Ministry of Health of Indonesia, National Institute of Health Research and Development, Jakarta, Indonesia; 3 Eijkman-Oxford Clinical Research Unit, Jakarta, Indonesia; 4 Centre for Tropical Medicine, Faculty of Medicine, Public Health and Nursing, Universitas Gadjah Mada, Yogyakarta, Indonesia

**Keywords:** Sub-national disparities, Anti-malarial drug, Eastern Indonesia, The 2018 Indonesia basic health survey, Public health

## Abstract

**Background:**

Poor access to health care providers was among the contributing factors to less prompt and ineffective malaria treatment. This limitation could cause severe diseases in remote areas. This study examined the sub-national disparities and predictors in accessing anti-malarial drug treatment among adults in Eastern Indonesia.

**Methods:**

The study analyzed a subset of the 2018 National Basic Health Survey conducted in all 34 provinces in Indonesia. We extracted socio-demographic data of 4655 adult respondents diagnosed with malaria in the past 12 months in five provinces in Eastern Indonesia. The association between socio-demographic factors and the access to anti-malarial drug treatment was assessed using logistic regression.

**Results:**

Over 20% of respondents diagnosed with malaria within last 12 months admitted that they did not receive anti-malarial drug treatment (range 12–29.9%). The proportion of untreated cases was 12.0% in East Nusa Tenggara, 29.9% in Maluku, 23.1% in North Maluku, 12.7% in West Papua, and 15.6% in Papua. The likelihood of receiving anti-malarial drug treatment was statistically lower in Maluku (adjusted OR = 0.258; 95% CI 0.161–0.143) and North Maluku (adjusted OR = 0.473; 95% CI 0.266–0.840) than those in Eastern Nusa Tenggara (reference). Urban respondents were less likely to receive malaria treatment than rural (adjusted OR = 0.545; 95% CI 0.431–0.689).

**Conclusions:**

This study found that there were sub-national disparities in accessing anti-malarial drug treatment in Eastern Indonesia, with a high proportion of untreated malaria cases across the areas. Findings from this study could be used as baseline information to improve access to anti-malarial drug treatment and better target malaria intervention in Eastern Indonesia.

## Background

Malaria elimination strategy requires strong early diagnosis capacity and effective prompt treatment [1]. However, delivering prompt treatment can be particularly challenging in rural areas with poor access to anti-malarial drug treatment [2]. As one of Southeast Asia’s countries with the highest malaria burden and varied epidemiological and entomological landscape [3, 4], Indonesia is complicated by high contribution of malaria from rural settings in Eastern provinces. Although most districts in western and central Indonesia have been certified malaria-free in 2020, five provinces in Eastern Indonesia i.e., Papua, West Papua, Maluku, North Maluku, and East Nusa Tenggara contributed to over 60% of total malaria cases in Indonesia [5]. Forest ecosystem and mobility factors (mobile and migrant population) added the complexity of malaria problem in these settings [6].

Indonesia is aiming to achieve malaria elimination by 2030 [7]. In order to improve malaria treatment access, the Indonesian government has implemented the early detection and administration of anti-malarial drugs by empowering local malaria cadres, especially in areas with limited access to health service facilities, lack of health staff, and medical supplies [8]. However, while the majority of provinces (25/34) in the western and central regions of Indonesia has achieved ACT coverage above 90%, the Eastern region has not achieved the targeted coverage, particularly in Maluku (74,7%) and ENT (80,1%) [9].

Previous studies documented varied factors that affect health services accessibility, malaria awareness and attitudes, and socioeconomic characteristics [10–15]. Distance to health facility, limited transportation to reach rural and remote areas, self-medication, low treatment rate, limited availability to health staff and medical supplies, are responsible to the malaria problems [16–18]. National Malaria Information System reported provinces in Eastern Indonesia had lower target coverage of malaria treatment than in Western Indonesia. This study assessed sub-national disparities and predictors in accessing anti-malarial drug treatment in five provinces in Eastern Indonesia.

## Methods

### Study sites

The study limited analysis to five provinces in Eastern Indonesia that had a high malaria annual parasite incidence (API) (Maluku, API = 1,16‰; North Maluku 0,39‰; East Nusa Tenggara 3,42‰; West Papua 8,49‰; and Papua 52,99‰) [5, 9]. These areas were chosen as representative of high endemic areas of Indonesia, which contributed to 91.68% (*n* = 165,217) of Indonesia’s national malaria cases in 2017 (2018 Indonesia Health Profile).

The province of Maluku consists of 11 districts (54,185 km^2^), with a population of 1.5 million people [19]. North Maluku consists of 10 districts (31,982 km^2^), with a population of around 1,038,087 people [20]. The ENT consists of 22 districts (47,931 km^2^), with approximately 4.6 million inhabitants [21]. West Papua consists of 13 districts (99,671 Km^2^), populated by over 800,000 people [22]. Papua has 29 districts (316,553 km^2^), with a population of 2.8 million people [23].

### Study design and data collection

This study was a secondary analysis of the 2018 Indonesia Basic Health Survey conducted by the National Institute of Health Research and Development (NIHRD), Ministry of Health of Indonesia. The survey was a community-based cross-sectional survey conducted in 34 provinces, from April to May 2018. This survey aimed to assess public health indicators key for policymaking at national, provincial, and district-level. The present study employed a subset of the 2018 Indonesia Basic Health Survey data. Sample were collected using a systematic random sampling model and a probability proportional to size approach by stratified multi-stage sampling. Data collection was carried out by around 10,000 trained enumerators with a minimum qualification of health graduate diploma. The enumerators were trained on how to obtain written informed consent, conducting interview, and completing questionnaires and other data collection forms. During data collection, the enumerators were supervised by at least one designated field coordinator employed at each district. These enumerators visited selected households accompanied by local health authorities and village leaders. The study followed informed consent protocols, and the enumerators collected information using standardized questionnaires via face-to-face interviews. The questionnaire consisted of several sections, including the household level section and individual-level section. Malaria was one of the infectious diseases included in the questionnaire.

In particular, the 2018 Indonesia Basic Health Survey surveyed 295,720 households with 1,091,528 household members in 34 provinces; for this analysis, a subset of data from five selected provinces were analyzed. The analysis was restricted to samples aged ≥15 years for this study (*n* = 4665); we chose this as it corresponds to the age group within the age range defined as an adolescent in Indonesia [24].

### Data source

The data source for this study was the 2018 Indonesia Basic Health Survey conducted by the National Institute of Health Research and Development (NIHRD), Ministry of Health of Indonesia. We restricted our analysis among ≥15 years old individuals (*n* = 4665) in five provinces: Papua (*n* = 2876), West Papua (*n* = 558), Maluku (*n* = 147), North Maluku (*n* = 121) and East Nusa Tenggara (*n* = 963).

### Variables

The study’s variables were measured by interview using the standardized questionnaire used in the Indonesia Basic Health Survey series, published in reports and scientific articles [5, 25]. In this analysis, the outcome variable was anti-malarial drug treatment. The variable is coded as “1” if a household member received an anti-malarial drug, i.e., he/she has been given the anti-malarial medication (ACT 3 days + Primaquin 1 day or taking anti-malarial drug ACT 3 days + Primaquin 14 days) after diagnosed in the past 12 months by local health care providers/physicians as having positive laboratory-confirmed malaria before the survey; and “0” if he/she reported otherwise. The answer to this question was binary: code 1 (Yes) and code 0 (No). Malaria has typically been confirmed in health facilities using Rapid Diagnostic Tests (RDTs) and microscopy. For this study, the interviewer carried out no screening tests. The study construct variables based on respondents’ recall of whether they have been given anti-malarial drugs after laboratory diagnosed malaria in the past 12 months.

The explanatory variables were age (grouped into two categories: 15–24, and more than 24 years), gender (male/female), place of residence (urban/rural), marital status (single/married), education level (did not complete primary education, completed primary, secondary or tertiary education). Occupation was categorized into three: (1) unemployed, including students; (2) farmer, including farmer, fisherman; and (3) non-farmer, including civil employee, private employee, entrepreneur, labor/driver/housemaid. Wealth status (poorest, poorer, middle, richer, and richest), access to health insurance (Yes/No) and transportation cost (≤ Rp. 15,000/ > Rp. 15,000).

### Statistical analysis

At the initial stage of the analysis, the study performed a bivariate analysis using the chi-square test to see differences in accessing anti-malarial drug treatment and respondents’ sociodemographic between provinces. In the next stage, the study tests all variables using a collinearity test to ensure no symptoms of a strong relationship between the independent variables. At the final stage, the study employed a logistic regression because of the nature of the dependent variable. The author performs all statistical analyzes by SPSS 22 software. The study generated a map of the geographical distribution of anti-malarial drug treatment distribution in ArcGIS 10.5 (ESRI Inc., Redlands, CA, USA). The author obtained the shapefile of administrative boundary polygons from the Bureau of Statistics of Indonesia) (http://www.silastik.bps.go.id).

## Results

**Fig. 1:**
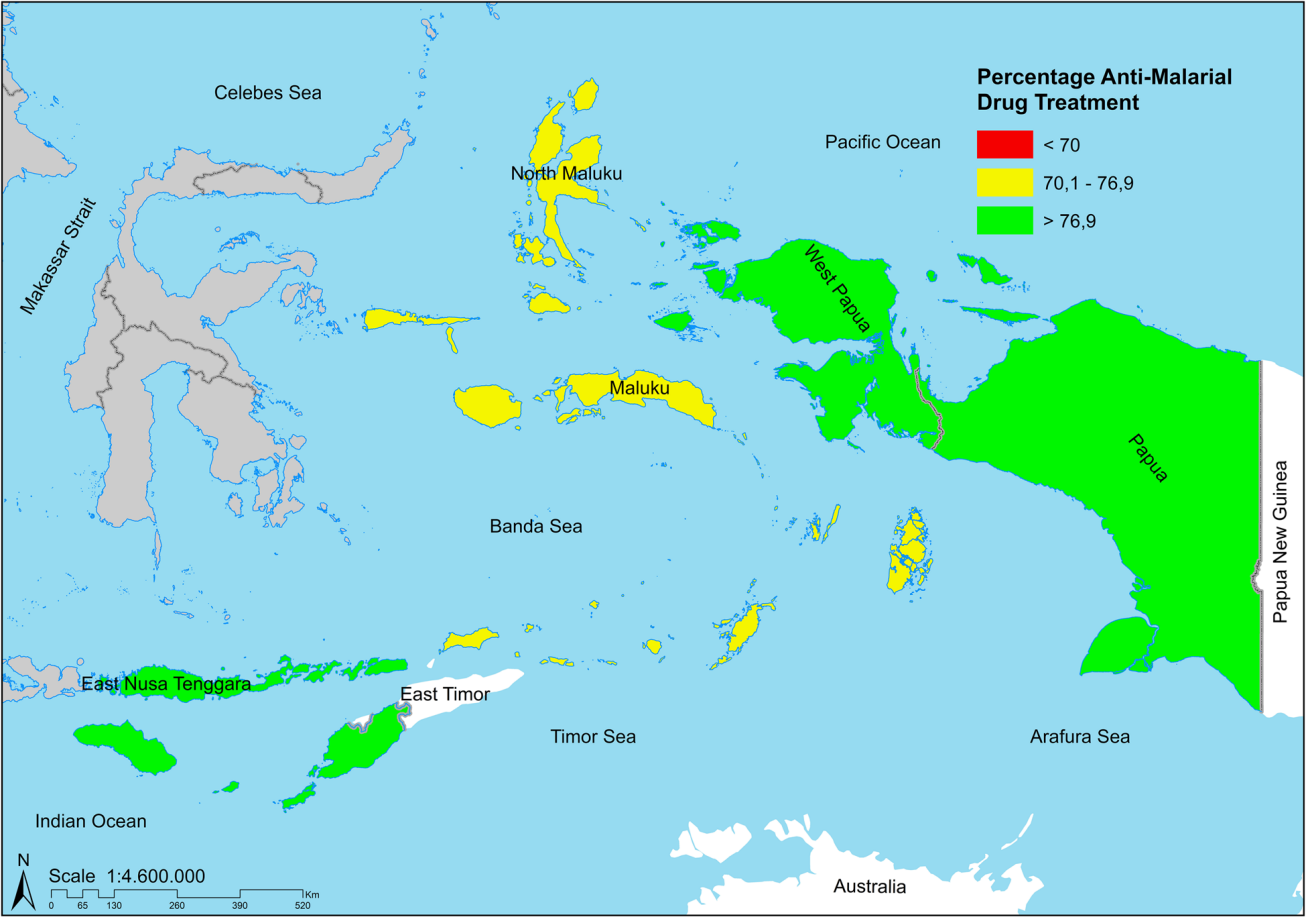
The geographic distribution of anti-malarial drug treatment distribution among adults ≥15 years old in eastern Indonesia (see Table 1 for values). The map depicted in the image belongs to the author. The map is generated using ArcGIS 10.5 (ESRI Inc., Redlands, CA, USA). The author obtained the shapefile of administrative boundary polygons from the Bureau of Statistics of Indonesia) (http://www.silastik.bps.go.id)

Figure 1 presents the spatial distribution of anti-malarial drug treatment in eastern Indonesia, including Maluku, North Maluku, East Nusa Tenggara (ENT), West Papua, and Papua Province. The proportion of respondents who did not receive anti-malarial treatment was 12.0% in East Nusa Tenggara, 29.9% in Maluku, 23.1% in North Maluku, 12.7% in West Papua, and 15.6% in Papua (Table 1).

**Table 1:** Descriptive statistic socio-demographic of the respondent in accessing anti-malarial drug treatment by the province in Eastern Indonesia (*n* = 4665)

Characteristics	Region/Province										***P***
	East Nusa Tenggara		Maluku		North Maluku		West Papua		Papua		
	n	%	n	%	n	%	n	%	n	%	
Malaria treatment											
No	116	12.0%	44	29.9%	28	23.1%	71	12.7%	448	15.6%	< 0.001
Yes	847	88.0%	103	70.1%	93	76.9%	487	87.3%	2428	84.4%	
Place of residence											
Urban	128	13.3%	45	30.6%	29	24.0%	185	33.2%	857	29.8%	< 0.001
Rural	835	86.7%	102	69.4%	92	76.0%	373	66.8%	2019	70.2%	
Age (mean)	963	28.10	147	27.48	121	30.12	558	27.43	2876	27.79	< 0.001
Gender											
Male	463	48.1%	90	61.2%	75	62.0%	313	56.1%	1509	52.5%	0.001
Female	500	51.9%	57	38.8%	46	38.0%	245	43.9%	1367	47.5%	
Marital status											
Single	540	56.1%	74	50.3%	54	44.6%	293	52.5%	1412	49.1%	0.002
Married	423	43.9%	73	49.7%	67	55.4%	265	47.5%	1464	50.9%	
Education level											
No education	78	9.0%	3	2.2%	6	5.3%	50	10.1%	380	14.7%	< 0.001
Primary	631	73.1%	79	57.7%	86	76.1%	273	55.4%	1486	57.4%	
Secondary	114	13.2%	42	30.7%	17	15.0%	121	24.5%	556	21.5%	
Higher	40	4.6%	13	9.5%	4	3.5%	49	9.9%	168	6.5%	
Occupation											
Unemployed	122	12.7%	28	19.0%	15	12.4%	113	20.3%	562	19.5%	< 0.001
Farmer	315	32.7%	43	29.3%	44	36.4%	88	15.8%	740	25.7%	
Non-farmer	526	54.6%	76	51.7%	62	51.2%	357	64.0%	1574	54.7%	
Wealth status											
Poorest	455	51.6%	25	23.1%	14	16.1%	137	34.0%	814	34.0%	< 0.001
Poorer	173	19.6%	30	27.8%	24	27.6%	34	8.4%	280	11.7%	
Middle	142	16.1%	28	25.9%	25	28.7%	69	17.1%	349	14.6%	
Richer	72	8.2%	16	14.8%	17	19.5%	67	16.6%	402	16.8%	
Richest	39	4.4%	9	8.3%	7	8.0%	96	23.8%	548	22.9%	
Health insurance											
No	96	10.0%	12	8.2%	9	7.4%	33	5.9%	102	3.5%	< 0.001
Yes	867	90.0%	135	91.8%	112	92.6%	525	94.1%	2774	96.5%	
Transportation cost											
≤ Rp. 15,000,-	619	64.3%	111	75.5%	84	69.4%	451	80.8%	1846	64.2%	< 0.001
> Rp. 15,000,-	344	35.7%	36	24.5%	37	30.6%	107	19.2%	1030	35.8%	

### Characteristic of respondents

Table 1 provides the socio-demographic characteristics of surveyed respondents. Most of respondents lived in rural areas and also received anti-malarial drugs. Respondents in the North Maluku had slightly older than respondents in the other areas. Malaria was more prevalent in males at all provinces, except in the East Nusa Tenggara. Higher proportion of respondents was observed with primary education background. Patients as farmers and un-employed patients contributed about a half of total respondents. Over 50% of respondents reported poor wealth status. Most of respondents had health insurance. High proportion of respondents mentioned the they spent transportation costs to nearest health facility less than 15,000 IDR (amount 1 $ US).

### Factors associated with anti-malarial treatment

Table 2 shows the final logistic regression model assessing factors associated with access to anti-malarial drug treatment in Eastern Indonesia. The model shows that malaria patients in Maluku and North Maluku were less likely to have anti-malarial drug treatment than those patients in East Nusa Tenggara (ENT), adjusted OR (aOR) 0.258 (95% CI 0.161–0.413), and 0.473 (95% CI 1.266–0.840), respectively. Moreover, there was no significant difference in proportion of patients who have anti-malarial drug treatment in West Papua (aOR 0.951; 95% CI 0.648–1.396), and Papua (aOR 0.894; 95% CI 0.682–1.172), compared to that in ENT.

**Table 2:** Logistic regression of anti-malarial treatment among malaria patients in Eastern Indonesia (*n* = 4665)

Predictor	Malaria Treatment	
	adjusted OR (95%CI)	***p***-value
Region/Province		
East Nusa Tenggara	1 (reference)	–
Maluku	0.258 (0.161–0.413)	< 0.001
North Maluku	0.473 (0.266–0.840)	0.011
West Papua	0.951 (0.648–1.396)	0.798
Papua	0.894 (0.682–1.172)	0.418
Place of Residence		
Urban	0.545 (0.431–0.689)	< 0.001
Rural	1 (reference)	–
Age	0.996 (0.988–1.003)	0.242
Gender		
Male	0.978 (0.796–1.201)	0.831
Female	1 (reference)	–
Marital status		
Single	1 (reference)	–
Married	0.838 (0.641–1.096)	0.197
Education level		
No education	1 (reference)	–
Primary	0.785 (0.552–1.116)	0.178
Secondary	0.973 (0.624–1.408)	0.755
Higher	1.021 (0.609–1.714)	0.937
Occupation		
Unemployed	1 (reference)	–
Farmer	1.381 (1.003–1.903)	0.048
Non-farmer	1.042 (0.792–1.371)	0.768
Wealth status		
Poorest	1 (reference)	–
Poorer	0.614 (0.451–0.836)	0.002
Middle	1.033 (0.743–1.438)	0.845
Richer	0.630 (0.463–0.857)	0.003
Richest	0.742 (0.545–1.010)	0.058
Health insurance		
No	1 (reference)	–
Yes	0.828 (0.503–1.361)	0.456
Transport cost		
≤ Rp. 15,000,-	0.820 (0.657–1.023)	0.078
> Rp. 15,000,-	1 (reference)	–

The multivariable analysis also found that place of residence, occupation, and wealth status were associated with anti-malarial drug treatment in Eastern Indonesia. The odd of having anti-malarial drug treatment was lower for malaria patients living in urban areas compared to those living in rural (aOR 0.545; 95% CI 0.431–0.689). Patients whose occupation as farmers were more likely to have anti-malarial drug treatment than those who were unemployed (OR 1.381; 95% CI 1.003–1.903). The poorer and richer patients were less likely to have anti-malarial drug treatment than the poorest, aOR 0.614 (95% CI 0.451–0.836), and 0.630 (95% CI 0.463–0.857), respectively. There was no significant association between age, gender, marital status, education level, health insurance, and transport cost to health facility, with access to anti-malarial drug treatment in Eastern Indonesia.

## Discussion

This study described the analysis of subset data from a nationwide community-based, cross-sectional survey conducted in Indonesia in 2019 (the 2018 Indonesia Basic Health Survey). The study revealed geographical variations and factors associated with access to anti-malarial drug treatment among malaria patients living in high endemic settings in the Eastern Indonesia. The odd of having anti-malarial drug treatment was associated with residence, occupation, and wealth status. There were no associations between age, gender, marital status, education level, health insurance, and transportation cost to health facility, with access to anti-malarial drug treatment in the Eastern Indonesia.

Malaria patients in ENT, Papua, West Papua, were more likely to access anti-malarial drug treatment than those patients in Maluku and North Maluku. Interestingly, in these areas, there were three provinces with the higher proportions of patients without any formal education, and with poorest wealth status. Thus, the findings contrast with other previous research findings concluding that lower education and economic status were associated with lower access to anti-malarial drug treatment. This potentially reflects other factors could have more impact on increasing access to anti-malarial drug treatment but were not assessed in this study. For examples, disparities in mass malaria elimination campaign, the availability of the malarial drugs at the primary healthcare facilities level, and the potential role of research institutions and local non-government organizations [26–28].

This study also revealed that the odd of having anti-malarial drug treatment was higher among farmers. A possible explanation for this is because farming work is often associated with higher exposure to malaria in many malaria-endemic areas in Indonesia due to doing night activities in farms, plantations, or forests [25, 29–32]. The farmer patients in this study could have a history of malaria infection and treatment, which made them more likely to notice symptoms and seek for treatment earlier than non-farmers [33]. A previous study in Papua found that farmers left their homes after daybreak, between 4:00 a.m. until 5:00 p.m., which is still within the range of an active hour for Anopheles feeding habits and puts them at risk of transmission [34, 35]. Furthermore, findings in Aceh reported since many rice fields in Indonesia were located far from home, some farmers stayed in the rice fields for several days. Farmers who were unable to return home every night stayed at forest suburban tree plantations for several nights to 2 weeks. Even more, they could spend up to 1 month in plantations located further in the forest, partially to work and partially to protect their crops from animals. During this period, farmers slept in simple huts [29].

Our present study found residence, occupation, and wealth status as significant predictors of anti-malarial drug treatment among malaria patients in the Eastern Indonesia. Similar studies also reported that malaria is generally prevalent in rural communities [36, 37], including several previous studies in Indonesia [25, 30, 38]. This condition could contribute to disparities between urban and rural areas in access to anti-malarial drug treatment among malaria patients [39, 40]. Additionally, it will get worse as the majority of Indonesians have difficulty getting proper healthcare [41]. Based on the 2018 Indonesia Basic Health Survey, knowledge of access to health facilities varied widely among residence types. Households in rural areas had difficulty in accessing hospitals (rural: 43% vs. urban: 13%) and primary health centers (rural: 36.8% vs. urban 22.5%) than households in urban [42].

Furthermore, the present study also revealed wealth status was associated with disparities in access to anti-malarial drug treatment among malaria patients. Consistent with this finding, previous research explained the emphasis of interventions on the most disadvantaged groups was likely to be more effective in addressing disparities because of the association of malaria infection with low socioeconomic status [43]. It also explained the importance of approaching the problem solving through economic and social dimensions as well as epidemiological and geographic data as an analytical tool to help malaria control programs focus on vulnerable subpopulations [44]. Therefore, it is essential to strengthen the drug supply system to ensure the distribution and coverage of anti-malarial drug treatment. A previous study in rural Tanzania informed that information technology in reporting drug stocks (SMS for Life) was one type of the best practices in improving the efficiency and effectiveness of drug control. Besides that, regular counseling, exemption from medical expenses, and improved living environment also need to be provided [45].

Further analyses demonstrated that age, gender, marital status, education level, health insurance, and transportation cost were not associated with acquiring anti-malarial drug treatment among malaria patients in the Eastern Indonesia. Although the researchers found education level was not the factor, intriguing evidence identified that anti-malarial drug treatment was more likely acquired by the respondents with primary education level. A previous study in Tanzania identified higher education levels were associated with a higher score of preventive practices. Meanwhile, the level of education was not in-line with seeking treatment behavior. Moreover, access to care and seeking for treatment were influenced by several other variables and culture [46].

Other findings also found that the transportation cost was not correlated with the outcome variable. However, transportation was a determinant factor associated with access to health care facilities. A study in Gambia revealed that free regular access to transportation significantly reduced delays in health care facility [47]. This result may be partly due to the improvement in malaria control program in general, particularly in the Eastern Indonesia [48]. Several previous studies have shown that easier accessibility could increase the effectiveness of anti-malarial drug treatment coverage [41, 49, 50].

Another intriguing result in this study was that health insurance ownership was not associated to disparities in access anti-malarial drug treatment. The current research contradicts with a previous study that identified that proximity to health care facilities and access to health insurance were related to the use of care and treatment [51]. While it may not determine a single role, health insurance can minimize the disparity in access to health facilities between the rich and poor populations [52]. The converse results explained that multifactor levels i.e., individual, interpersonal, and institutional/structural levels influenced individual health behavior [53].

A previous study showed that one of the limitations of the Global Malaria Eradication Campaigns in the 1950s and 1960s was the belief that malaria eradication could be accomplished with a one-size-fits-all policy rather than adjusted strategies to local contexts [54]. According Oaks et al. [55], prevention strategies should include considerations relevant to community care-seeking behaviors to gain successful adoption of malaria control methods in endemic areas. For example, preference for a traditional birth attendant has resulted in collaboration between village midwives and birth attendants in some areas of Indonesia [56], including in ENT where ‘*Sembur*’ culture serves as a local wisdom in care of pregnant mother with malaria in Kupang [57]. This approach integrated traditional healers with health promotion and thus led to better involvement with formal health treatment. Therefore, a local wisdom approach like this may be more successful in addressing disparities in accessing anti-malarial drug treatment in Eastern Indonesia. Other example of local wisdom approach is indigenous leaders were engaged as village health volunteers (VHVs). VHVs have been highlighted as an important primary health-care provider in rural PNG, where they have diagnosed and treated acute malaria cases. VHVs are the most accessible health-care staff to residents, thus could be a cost-effective and practical strategy to expand anti-malarial access [58]. Local wisdom is wisdom passed down from generation to generation or seen as experience of some groups in organizing social life and adjusting it to the environment to meet social needs, especially in attitude and actions [59]. Interventions in health promotion that use respectful and relevant local wisdom methods may improve the penetration and durability of positive behavioral change. Best-practice health promotion can be accomplished by integrating local wisdom at any health promotion levels through the empowerment of local societies, their traditions, and certain environmental conditions [60].

Our study has certain limitations. First, this study is cross-sectional, so that we could not infer the causal relationships, the gaps found and revealed in this study are still limited to a depthless understanding. Second, we should note that malaria cases of this study were self-reported malaria. There were no diagnostic tests to confirm malaria infection among participants. Third, children were not included in this study, regardless of the fact that malaria is a major concern not only for adults but also for children under the age of five. Therefore, we should cautiously interpret the results as this could lead to biases (e.g., reporting bias and social desirability bias).

## Conclusions

The study concludes there were sub-national disparities in accessing anti-malarial drug treatment in Eastern Indonesia. Moreover, the study also found that regions, places of residence, occupation, and wealth status were predictors in accessing anti-malarial drug treatment. Findings from this study could be used as baseline information to improve access to anti-malarial drug treatment and better target malaria intervention in Eastern Indonesia.

## Data Availability

The data that support the findings of this study are available from the Data Management Laboratory of the National Institute of Health Research and Development (NIHRD), the Ministry of Health of the Republic of Indonesia under license and so cannot be made freely available. Data can be made available after approval of a written request to the Data Management Laboratory—NIHRD (via mandat@litbang.depkes.go.id/labmandat.litbangkes@gmail.com).
